# Quality of information offered to women by drug sellers providing medical abortion in Nigeria: Evidence from providers and their clients

**DOI:** 10.3389/fgwh.2022.899662

**Published:** 2022-08-17

**Authors:** Akanni Akinyemi, Onikepe Oluwadamilola Owolabi, Temitope Erinfolami, Melissa Stillman, Akinrinola Bankole

**Affiliations:** ^1^Center for Research, Evaluation Resources, and Development, Abuja, Nigeria; ^2^Department of Demography and Social Statistics, Obafemi Awolowo University, Ife, Nigeria; ^3^Vital Strategies, New York, NY, United States; ^4^Guttmacher Institute, New York, NY, United States

**Keywords:** misoprostol, drug sellers, medical abortion, information, quality of care, Nigeria

## Abstract

**Background:**

Evidence confirmed that the demand for medical abortion (MA) increased significantly during the COVID-19 outbreak in many developing countries including Nigeria. In an abortion-restrictive setting like Nigeria, local pharmacies, and proprietary patent medicine vendors (PPMVs) continue to play a major role in the provision of MA including misoprostol. There is the need to understand these providers' knowledge about the use of misoprostol for abortion and the quality of information they provide to their clients. This analysis is focused on assessing the quality of care provided by both drug seller types, from drug sellers' and women's perspectives.

**Methodology:**

This study utilized primary data collected from drug sellers (pharmacists and PPMVs) and women across 6 Local Government Areas in Lagos State, Nigeria. The core sample included 126 drug sellers who had sold abortion-inducing drugs and 386 women who procured abortion-inducing drugs from the drug sellers during the time of the study. We calculate quality-of-care indices for the care women received from drug sellers, drawing on WHO guidelines for medication abortion provision. The index based on information from the sellers had two domains—technical competency and information provided to clients, while the index from the women's perspectives includes an additional domain, client experience.

**Results:**

Results show that the majority of drug sellers in the sample, 56% (*n* = 70), were pharmacists. However, far more than half of women 60% (*n* = 233) had visited PPMVs. Overall, the total quality score amongst all drug sellers (mean 0.48, SD0.15) was higher than the total score calculated based on women's responses (mean 0.39, SD 0.21). Using our quality-of-care index, pharmacies and PPMVs seem to have similar technical competency (mean score of 0.23, SD 0.13 in both groups (range 0–1), whilst PPMV's performed better on the information provided to client domain (mean score of 0.79, SD 0.17 compared with pharmacies 0.69, SD 0.25). Based on women's reports, PPMVs scored better on both quality of care domains (technical competency and information provided to clients) compared with pharmacies.

**Program/Policy Implication:**

In resource-constrained settings such as Nigeria, particularly in the context of health emergencies like COVID-19, there is the need to continue to strengthen and engage PPMVs' capacity and skills in dispensing and administration of MA drugs as a harm reduction strategy. Also, there is the need to target frontline providers in pharmacies for training and skill upscale in MA provision.

## Introduction

Although there is limited documented evidence of its spread, the use of medical abortion (MA) to terminate pregnancies seems to be expanding in Sub-Saharan Africa (SSA) and drug vendors are expected to be important providers of these pills (1). Because of their accessibility and affordability, drug vendors are the preferred first source of medication, and often for health care overall, in the region (2, 3). Nigeria, with the largest population in Africa, has a restrictive abortion law, permitting abortions only to save the life of a woman. Nevertheless, they have a high rate of induced abortion—estimated at 33 per 1,000 women of reproductive age in 2012 (4). MA access through pharmacies has been on the rise. A small-scale study conducted in Nigeria in 2006 estimated that 3% of drug sellers reported selling misoprostol (5), while a 2018 hospital-based study of women admitted for postabortion care shows an increasing trend in women using MA compared with other methods (6). This suggests that a growing number of women are able to access MA in Nigeria.

Given the role of local pharmacies and proprietary patent medicine vendors (PPMVs) as confidants and the immediate go-to sources of health care in Nigeria and other Sub-Saharan African countries, these drug sellers are likely to play a major role in the provision of misoprostol in the community. Because of their proximity, flexibility, as well as privacy, and less expensive services they offer relative to health facilities, drug sellers' services are mostly preferred, including visiting their outlets for the sale of misoprostol, a common MA drug (1, 7, 8).

Medication abortion has been identified as partly responsible for the decline in severe abortion-related morbidity and mortality across the globe (9, 10). The safety and efficacy of MA when correctly administered, as well as its crucial role in mitigating the consequences of unsafe abortions, have been well documented (11–13). However, the use-effectiveness depends to a large extent on the levels of knowledge and understanding of PPMVs/Pharmacies, compliance to recommended new guidelines by WHO and in-country health care administrators, and the level of information provided to women at the point of purchase. The WHO the new guidelines recommend that pharmacists/pharmacy workers can provide MA, in addition to other healthcare professionals In Nigeria, most pharmacies operate at sub-standard levels, with a growing proportion of operators functioning as medicine vendors and chemists (14). These medicine vendors operate at the subsistence level with the scarcity of qualified technical personnel and finances as common operational constraints (14). There is a general knowledge gap, including among drug sellers, in understanding issues around access to safe, self-managed medication abortion, and this situation was likely exacerbated during the pandemic (15). In Nigeria, a study among women who obtained misoprostol-containing medication from pharmacies and PPMVs for abortion in Lagos state reported a low level of knowledge and practical skill among these providers. For instance, they reported that drug sellers covered only three of nine items that the study considered necessary for successful self-management of abortion using misoprostol. About 35% reported being told that they could expect some cramping after taking the medication and only 13% reported being told about severe bleeding that could indicate a potential complication (16). In Senegal, with a similar legal context as Nigeria and where pharmacies and PPMVs are integral in supplying medications to the general public, (17) also found among the staff of pharmacies and PPMVs low levels of knowledge about misoprostol use, treatment regimens and side effects as well as low levels of training on the uses of the medication. This evidence suggests that adequacy and adherence to existing standard guidelines and protocols for providing medication abortion is less likely when MA is sought from patent medicine vendors. However, to improve women's health in the country, this situation can and must be mitigated through appropriate education and intervention.

The World Health Organization (WHO) has provided some clarity around safe abortion care and medication abortion provision guidelines. The updated guidelines for safe abortion, task sharing for abortion, and medication abortion recommend the task of conducting safe abortion and post-abortion care is within the purview of skilled healthcare professionals (midwives, nurses, and physicians) (18). The guidelines provide information on the timing, dosage, dosing intervals, and routes of administration of medications to manage abortion, as well as the timing of contraception initiation following a medication abortion. In Nigeria, where abortion seekers must obtain abortifacients covertly because of the restrictive laws, this responsibility has largely shifted to drug sellers (pharmacists and PPMVs) who do not necessarily possess the requisite training to manage abortion and post-abortion care. Evidence from a scoping review suggests that adhering to the quality-of-care indicators and the WHO safe abortion guidelines and protocol on medication abortion is still challenging to patent and medicine vendors (19). A recent assessment of the adequacy of information drug sellers provide to their abortion-seeking clients (16) revealed that basic instructions on drug use and administration were often provided, but inadequate information was provided on warning signs of complications and how to assess abortion completeness. The study (16) focused on women's reported experiences, whilst this current article compares women's reports with pharmacy reports, and different types of pharmacy workers.

The focus of this analysis is therefore to assess the quality of care provided by both drug seller types, from drug sellers' and women's perspectives. The specific objectives are to (1) assess whether the quality of care differs by type of drug sellers (pharmacists vs PPMVs) and (2) to explore the consistency between the drug sellers' and women's reported quality of care by drug seller type.

## Ethical approval

The National Health Research Ethics Committee in Nigeria and the Institutional Review Board of Guttmacher Institute, U.S.A. approved the study.

## Methodology

### Study design and sample

**Figure 1:**
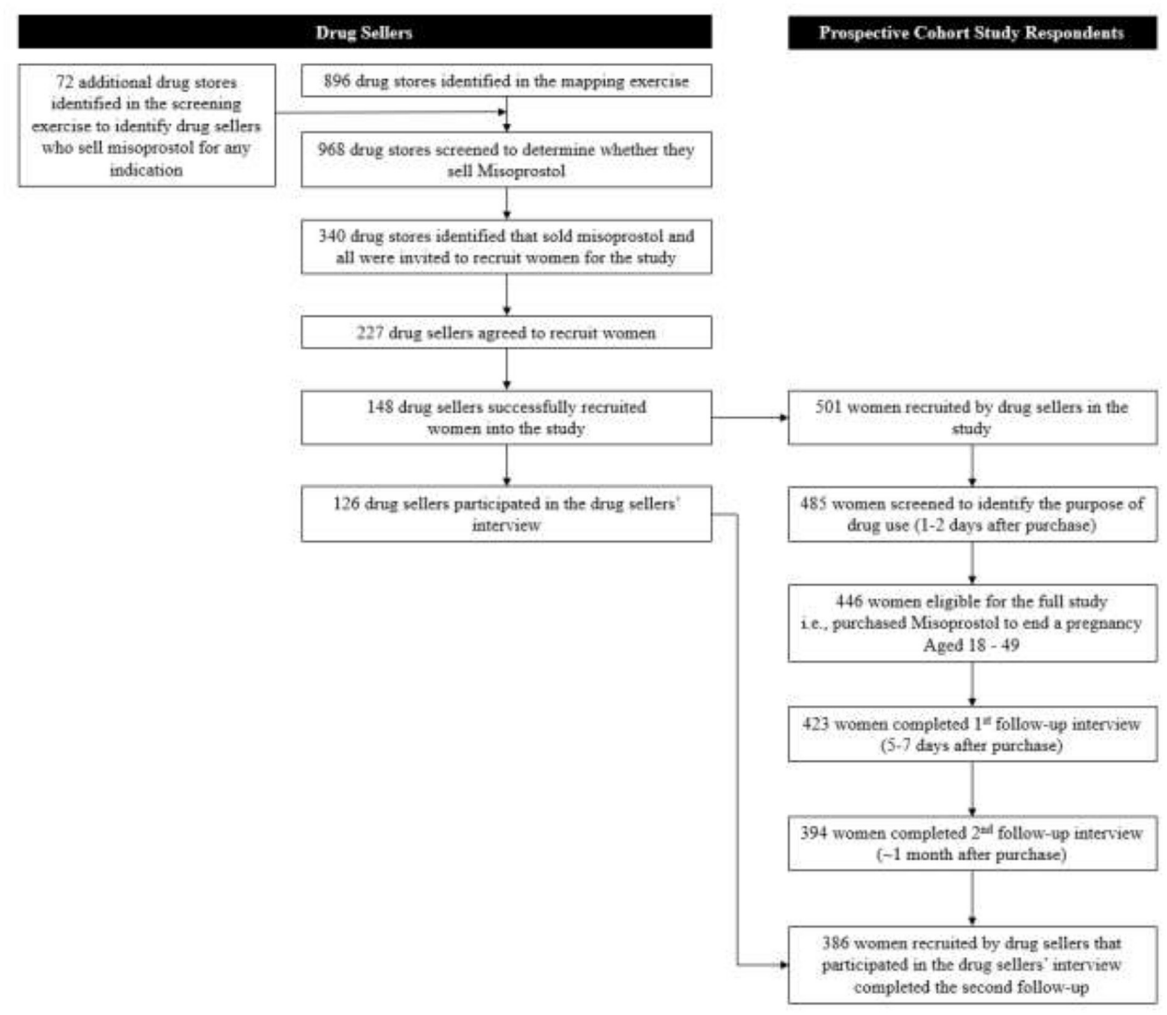
Sample of drug sellers, recruitment of women, and retention throughout the study.

This study is part of a larger prospective study that collected data from drug sellers and women aged 18 to 49 across 6 Local Government Areas in Lagos State, Nigeria (16). The sample included two categories of drug sellers [registered pharmacies and patent and proprietary medicine vendors (PPMVs)] who reported selling misoprostol-containing medication for any indication and women who procured these drugs from the drug sellers to terminate a pregnancy. In this analysis, we included 126 drug sellers who participated in a drug seller's survey and recruited women into the study and the 386 women they recruited who completed all three prospective interviews during the study. Figure 1 is a flowchart describing study enrollment and our final sample. Further details about the larger study's sample data collection and information obtained from the respondents are available in a previous publication (16).

### Quality of care measurement

For this analysis, we proposed quality of care (QoC) indicators within defined domains and computed indicator-specific QoC scores, an overall summary score for each domain, and a total QoC score drawing on the World Health Organization's guidelines for safe abortion, task sharing for abortion, and medication abortion guidelines (20, 21). We defined three broad domains of quality: technical competence, information given to clients, and client experience of care. Each domain is represented by at least one indicator and each indicator is informed by responses to at least one question from the women's survey and/or the drug seller's knowledge, attitude, and practice tool. We computed indicator-specific scores and domain scores based on women's responses to questions on their perspectives of the care they received and the drug sellers' responses about what they would theoretically do for a client seeking misoprostol to induce an abortion. Appendix Table 1 shows the domains, indicators, the corresponding questions, and summaries of the responses which were used to compute the scores. The full list of indicators from both perspectives is as follows: Within technical competence, we included pregnancy confirmation, gestational age or last menstrual period assessment, accurate drug dosage, accurate route of drug administration, and appropriate gestational age to use medications. Within information given to the client, we included information on the name of the medication, how to take the medications, side effects, risks and complications associated with a medication abortion, how to assess the completeness of termination, and actions clients can take to address complications. Client experience had one indicator: information provided on pain relief.

For some indicators (confirming completeness of termination and actions to address complications) there were no appropriate questions to evaluate quality available in the women's survey. Similarly, there was no question to evaluate the provision of pain relief in the drug sellers' survey. Each indicator within each domain was assigned a score of 1. If multiple questions were used to compute the score for an indicator, the score of 1 was split equally between the questions, and the relative contribution of the responses to the indicator score was measured. The scores for all questions associated with the indicator were then added up to derive the score for that indicator. For example, under the technical competence domain for drug sellers, the pregnancy confirmation indicator was defined using responses to two questions: whether they would ask the woman about their last menstrual period; whether they would ask if the women did a pregnancy test; and whether they know how to correctly calculate gestational age. For each drug seller, an affirmative to each of these three questions was assigned a score of 0.333 (i.e., 1 divided by 3), and the scores were added up to derive a total score ranging from 0 to 1 with 1 representing the maximum pregnancy confirmation score (see Table 1). The scores for all indicators under each domain were then added together and rescaled by dividing the sum by the number of indicators to arrive at a domain score which is a proportion between 0 and 1. Similarly, we generate a total QoC score from both the drug sellers' and women's perspectives by adding up the scores for the relevant domains and dividing them by the number of domains. For example, for women, we added up the scores for the three domains and divided the result by three to arrive at a women's overall QoC score with a value between 0 and 1.

**Table 1:** Indices of quality of care regarding misoprostol provision by drug sellers measured from drug sellers' and women's perspectives, Lagos State, Nigeria.

**Drug sellers**	**Pharm**	**PPMV**	**Total**	**Women**	**Pharm**	**PPMV**	**Total**
	***N** **=*** **70**	***N** **=*** **56**	***N** **=*** **126**		***N** **=*** **153**	***N** **=*** **233**	***N** **=*** **386**
	**Mean** **±SD**	**Mean** **±SD**	**Mean** **±SD**		**Mean** **±SD**	**Mean** **±SD**	**Mean** **±SD**
Pregnancy Confirmation	0.43 ± 0.29	0.45 ± 0.26	0.44 ± 0.28	Pregnancy Confirmation	0.57 ± 0.46	0.85 ± 0.32	0.73 ± 0.41
Prescribed optimal dosage of Miso (1st Trimester)	0.10 ± 0.22	0.12 ± 0.21	0.11 ± 0.22	Was told the optimal dosage of Miso	0.53 ± 0.50	0.58 ± 0.49	0.56 ± 0.50
Prescribed optimal dosage of Miso (2nd Trimester)	0.00 ± 0.00	0.01 ± 0.07	0.00 ± 0.04				
Prescribed optimal route for Miso	0.50 ± 0.26	0.50 ± 0.24	0.50 ± 0.25	Was told the optimal route of Miso	0.22 ± 0.41	0.16 ± 0.37	0.18 ± 0.39
Knew appropriate GA to use drugs	0.74 ± 0.44	0.84 ± 0.37	0.79 ± 0.41				
**Technical competence score (Raw)**	**1.78** **±0.85**	**1.92** **±0.74**	**1.84** **±0.81**	**Technical competence score (Raw)**	**1.31** **±0.71**	**1.59** **±0.71**	**1.48** **±0.73**
**Technical competence score (Transformed)**	**0.36** **±0.17**	**0.38** **±0.15**	**0.37** **±0.16**	**Technical competence score (Transformed)**	**0.44** **±0.24**	**0.53** **±0.24**	**0.49** **±0.24**
Provided clients with information on the use of medications	0.81 ± 0.39	0.98 ± 0.13	0.89 ± 0.32	Was told the name of the medicine by the drug seller	0.13 ± 0.34	0.25 ± 0.43	0.20 ± 0.40
Dosage/Route Instructions given	0.73 ± 0.41	0.84 ± 0.32	0.78 ± 0.38	Dosage/Route Instructions received	0.59 ± 0.49	0.65 ± 0.48	0.63 ± 0.48
Told clients about potential experience of drug	0.52 ± 0.29	0.54 ± 0.33	0.53 ± 0.31	Was told about the potential experience of drug	0.32 ± 0.32	0.36 ± 0.27	0.35 ± 0.29
Told clients how to confirm pregnancy is terminated	0.47 ± 0.50	0.53 ± 0.50	0.50 ± 0.50				
Told clients how to address complications	0.26 ± 0.44	0.20 ± 0.40	0.23 ± 0.42				
**Information given (Raw)**	**2.79** **±1.23**	**3.08** **±0.96**	**2.92** **±1.12**	**Information given (Raw)**	**1.05** **±0.74**	**1.26** **±0.61**	**1.18** **±0.67**
**Information given (Transformed)**	**0.56** **±0.25**	**0.62** **±0.19**	**0.58** **±0.22**	**Information given (Transformed)**	**0.35** **±0.25**	**0.42** **±0.20**	**0.39** **±0.22**
				**Experience of clients (pain relief)**	**0.33** **±0.47**	**0.25** **±0.43**	**0.28** **±0.45**
**Total drug seller QoC score**	**0.46** **±0.18**	**0.50** **±0.15**	**0.48** **±0.17**	**Total women QoC score**	**0.37** **±0.24**	**0.40** **±0.19**	**0.39** **±0.21**

### Statistical analysis of levels and patterns of quality of care

We describe the socio-demographic characteristics of both categories of drug sellers and those of the women who obtained services from them. Thereafter we first compare the QoC reported by drug sellers and that reported by women, according to type of drug sellers. Then, we compare the QoC reported by women to those reported by the drug sellers who recruited them, by type of drug sellers. To ensure that these scores are comparable, we limit the indicators we use in both scales to those with questions available in both the women's and drug sellers' surveys. For this reason, we are unable to include the client experience domain in this comparison. Thereafter we performed a one-way analysis of variance (ANOVA) to assess whether the mean QoC score based on women's perspective varies by women's sociodemographic characteristics. We conducted this analysis for all drug sellers combined and separately by type. We used Stata version 16.0 for the statistical analysis.

### Role of the funding source

The funder of the study had no role in study design, data collection, data analysis, data interpretation, or writing of the report. The corresponding author had full access to all the data in the study and had final responsibility for the decision to submit for publication.

## Results

Results show that the majority of drug sellers in the sample 56% (*n* = 70) were pharmacists (Table 2). Our drug seller respondents in pharmacies were more educated than their counterparts in PPMVs, with 61% in pharmacies having more than a secondary education compared to 41% in PPMVs. Also, pharmacies were more likely to have more than one staff member than PPMVs (93 vs. 39%, respectively) and employ staff with post-secondary health-related qualifications other than the respondent to the interviews (67 vs. 27% in PPMVs). On the other hand, PPMVs were more likely to report having received training in safe abortion care (16 vs. 3% pharmacies) and postabortion care (11 vs. 1%). About 60%, (*n* = 233) of women in our sample patronized PPMVs (Table 3). Women who sought medication for abortion from PPMVs were more likely to be in family business/farming or housewives compared with those who did so from pharmacies (34 vs. 19%), while those who patronized pharmacies were more likely to be students than their counterparts who visited PPMVs (18 and 10%, respectively). More women in our sample with higher than secondary education and those who had never married or cohabited obtained their abortion medication at pharmacies (48 vs. 31% and 50 vs. 42%, respectively). Furthermore, women who had a pregnancy test done were more likely to have done a self-administered test (45 and 36%, respectively, compared to having done the test with a doctor or at a laboratory.

**Table 2:** Characteristics of drug sellers who offered women misoprostol for abortion, Lagos State, Nigeria.

	**Pharmacy**	**PPMV**	**Total**
	***N** **=*** **70**	***N** **=*** **56**	***N** **=*** **126**
**Drug Seller's highest level of education**			
Some junior secondary school	0.0%	3.6%	1.6%
Some senior secondary school	4.3%	8.9%	6.3%
Completed secondary school	34.3%	42.9%	38.1%
Higher education	61.4%	44.6%	54.0%
**Drug Seller has a post-secondary health-related qualification**			
No	44.3%	71.4%	56.3%
Yes	55.7%	28.6%	43.7%
**Other staff members with post-secondary health-related qualification**			
No	32.9%	73.2%	50.8%
Yes	67.1%	26.8%	49.2%
**The respondent or their colleague has received training on safe abortion care**			
No	97.1%	83.9%	91.3%
Yes	2.9%	16.1%	8.7%
**The respondent or their colleague has received training on post-abortion care**			
No	98.6%	89.3%	94.4%
Yes	1.4%	10.7%	5.6%
**The staff strength of drug store**			
A Staff	7.1%	60.7%	31.0%
2 to 4 Staff	58.6%	35.7%	48.4%
5 or more staff	34.3%	3.6%	20.6%
Median staff strength (IQR)	4(2)	1(1)	2.5(3)
**Respondent has attended to a client seeking to purchase misoprostol in the past**			
No	4.3%	12.5%	7.9%
Yes	95.7%	87.5%	92.1%
**Respondent has attended to a client seeking to purchase misoprostol to terminate a pregnancy in the past**			
No	27.1%	28.6%	27.8%
Yes	72.9%	71.4%	72.2%
**Respondents or their colleagues have refused to sell clients medication to terminate a pregnancy or bring back a missed period**			
No	22.9%	28.6%	25.4%
Yes	77.1%	71.4%	74.6%
**Respondents refer clients to other places if they cannot provide medications to terminate a pregnancy or bring back period**			
No	70.0%	66.1%	68.3%
Yes	30.0%	33.9%	31.7%

**Table 3:** Characteristics of women who obtained misoprostol from drug sellers for abortion, Lagos State, Nigeria.

	**Pharmacy**	**PPMV**	**Total**
	***N** **=*** **153**	***N** **=*** **233**	***N** **=*** **386**
**Age**			
18–24	25.5%	21.9%	23.3%
25–29	33.3%	31.3%	32.1%
30–34	23.5%	23.2%	23.3%
35–39	11.8%	18.0%	15.5%
40–44	4.6%	5.2%	4.9%
45–49	1.3%	0.4%	0.8%
Median age (IQR)	27(8)	29(9)	28(8)
**Parity**			
no parity information	40.5%	44.2%	42.7%
no children	19.0%	19.3%	19.2%
1–2 children	27.5%	18.5%	22.0%
3+ children	13.1%	18.0%	16.1%
**Highest level of education completed**			
No schooling or incomplete primary	0.0%	0.9%	0.5%
Primary/Junior secondary school	5.9%	8.6%	7.5%
Senior secondary school	46.4%	59.2%	54.1%
Some higher education (or more)	47.7%	31.3%	37.8%
**Employment status**			
Work outside the home for pay	54.2%	49.8%	51.6%
Family business or subsistence farm	17.6%	28.8%	24.4%
Housewife	1.3%	5.2%	3.6%
Student	18.3%	9.9%	13.2%
Unemployed	8.5%	6.4%	7.3%
**Marital status**			
Currently married or cohabiting	47.7%	51.9%	50.3%
Separated/divorced/widowed	2.6%	6.0%	4.7%
Never married/never cohabited	49.7%	42.1%	45.1%
**Has previously attempted to end a prior pregnancy**			
No	96.7%	93.6%	94.8%
Yes	3.3%	6.4%	5.2%
**Type of pregnancy test taken to confirm pregnancy**			
Confirmation *via* a test with a doctor	11.1%	16.7%	14.5%
Confirmation *via* test at a laboratory	9.2%	9.4%	9.3%
Self-administered urine test	45.1%	36.1%	39.6%

Table 1 presents the total, domain, and indicator-specific QoC scales from the perspectives of both drug sellers and women. From the drug sellers ' perspectives, the total quality of care score was higher in PPMVs than in pharmacies (mean 0.50, SD 0.15, and mean 0.46, SD 0.18 respectively), and the patterns in women's scores were similar. Overall, the total quality score amongst all drug sellers (mean 0.48, SD 0.17) was higher than the total score calculated based on women's responses (mean 0.39, SD 0.21). According to the drug sellers' perspectives, technical competence scores were low (mean scores 0.37, SD 0.16 out of a total of 1), but similar between PPMVs and pharmacies (mean 0.38, SD 0.15, and mean 0.36, SD 0.17, respectively). PPMVs were more likely to report knowing the appropriate gestational age to use drugs (mean 0.84, SD 0.37 vs. mean 0.74, SD 0.44, respectively). The scores in the information domain were higher than those for technical competence for both types of drug sellers. However, PPMVs reported better information given to clients than pharmacies (mean 0.62, SD 0.19 vs. mean 0.56, SD 0.25 respectively). PPMVs reported more accurate responses on telling clients how to use medications and the dosage and route of administration. However, pharmacies more commonly reported telling clients how to address complications than PPMVs (mean 0.26, SD 0.44 vs. mean 0.20, SD 0.40, respectively).

The QoC scores based on women's perspectives were low on each domain but showed a similar pattern to the scores based on drug sellers' perspectives. Women who obtained care from PPMVs reported greater technical competence (mean 0.53, SD 0.24 vs. mean 0.44, SD 0.24 in pharmacies) and received more information than those who visited pharmacies (mean 0.42, SD 0.20 vs., mean 0.35, SD 0.25, respectively). At the indicator level under technical competence, QoC from the women's perspectives was higher than from drug sellers' perspectives for pregnancy confirmation (mean 0.73, SD 0.41 vs. mean 0.44, SD 0.28) and prescribed optimal dosage of misoprostol for first-trimester abortion (mean 0.56, SD 0.50 vs. mean 0.11, SD 0.22). However, the score for the prescribed optimal route for misoprostol was lower according to women's perspectives (mean 0.18, SD 0.39 vs. mean 0.50, SD 0.25). On the other hand, under the information domain, the quality of care for all three relevant indicators was higher based on drug sellers' responses compared to women's perspectives. For example, according to drug sellers, the results for information on the use of medication were higher for pharmacies (mean 0.89, SD 0.32) compared to PPMVs (mean 0.20, SD 0.40). Yet, according to the women, pharmacies were more likely to recommend pain relief to them than PPMVs (mean 0.33, SD 0.47 and mean 0.25, SD 0.43, respectively).

When we limit the computation of the QoC scores based on the drug sellers' perspectives to include only indicators with parallel questions available within the women's scale, there is no change from the pattern of results reported above. However, there was a reduction in technical competence scores and an increase in the information given to client scores for both categories of drug sellers (Appendix Table 2). Compared to the scores obtained based on women's responses, the technical competence scores based on the reduced number of indicators for the drug sellers were much lower than previously noted above, overall, and for both types of drug sellers. For example, the new score for all drug sellers based on drug sellers' perspectives was (mean 0.23, SD 0.13) compared to (mean 0.49, SD 0.24) from women's perspectives. On the other hand, for information given, the new scores based on drug sellers' perspectives, for both groups, were much higher than previously observed. Based on their responses, the new score for all drug sellers is (mean 0.73, SD 0.22) compared to (mean 0.39, SD 0.22) from women's perspectives.

Appendix Table 3 shows the results of the one-way ANOVA. There appeared to be very few significant associations between sociodemographic characteristics of women and the quality of care they experienced. Looking at all drug sellers, there was a statistically significant difference in mean examination scores between at least two groups of women by employment status (*F* = 4.04, *p* = 0.003).

## Discussion of findings

Our findings show that women are more likely to obtain MA at PPMVs than at pharmacies. This is in line with existing evidence that shows that PPMVs act as the first point of care for health-seeking including for family planning and other reproductive health services among the majority of the Nigerian population, particularly in rural and lower-income communities (3, 22, 23). Our findings also suggest that there are differences in the socioeconomic characteristics of women who obtain care from PPMVs compared with pharmacies. MA drugs are usually paid for out-of-pocket by women procuring them. Thus, we hypothesize that women who may have lower purchasing power or prefer to pay less for MA drugs, such as housewives, workers in a family business, and farmers would be more likely to visit PPMVs than pharmacies. Furthermore, smaller PPMV stores are located in residential neighborhoods and are less likely to request to see formal prescriptions before dispensing medications, compared with large, registered pharmacies (24–26). On the contrary women with higher education status, which demonstrates a greater level of autonomy, are more likely to patronize pharmacies for abortion medications. In general, our results show that pharmacies in most communities in Lagos have employees that are better educated and more likely to have a health-related qualification when compared with PPMVs (5, 6). However, more staff of PPMVs reported on-the-job training, particularly in safe abortion and post-abortion care suggesting that more programmatic interventions have been carried out with this group of providers (8). Evidence from a scoping review (2) confirmed the importance of exposure to health-related training among PPMV, its impact on their service delivery, and the role of their association in periodically organizing training for them.

Comparing the QoC outcomes for PPMV and Pharmacies from both women's and drug sellers' perspectives yields interesting results. The similar levels of QoC (overall and for each considered domain) found among both groups of drug sellers further underscores the importance of the on-the-job training that the PPMVs received. The training reported appears to compensate for the lower level of education and formal health training reported by PPMVs which we initially hypothesized would result in poorer technical knowledge about MA and prescription skills. Several intervention studies across low- and middle-income countries, including Nigeria, point to the effectiveness of such on-the-job training concerning QoC (27, 28). With greater evidence of multiple staff members working in large, registered pharmacies, it is also possible that respondents to our survey—the frontline providers who women would encounter upon entering—are not the trained pharmacists themselves, but staff with limited clinical skills and technical knowledge. Facilitating frequent on-the-job training for front desk staff at drug stores, in addition to managers or registered pharmacists, is thus necessary to provide the support they need to provide medications like MA and other SRH commodities.

This is particularly critical as the role of drug sellers evolve and they are utilized more frequently for frontline SRH care (29), with the growing preference of people for quick and convenient access to health care within their community and amidst overburdened health facilities (which came more important during the COVID-19 pandemic), and with greater access to reproductive self-care commodities and services (30). Similar to a study in India (31), there is a knowledge-practice gap in the care drug sellers reported providing and women reported experiencing. Whilst we advocate for on-the-job training to improve drug sellers' technical competence, it is also important that interventions find the right incentives to bridge the gap between what drug sellers know and their consistency in providing essential information to women obtaining MA from them to optimize women's experiences of care.

The higher QoC score from the drug sellers' perspective compared with women in the information-provided domain, and the reversed case in the technical competence domain is noteworthy. The potential reason for these findings may not be unconnected with the experience and/or expectation of both groups on the subject. For instance, women with limited skills and knowledge about MA might be more likely to have overstated the technical competence of the drug sellers. On the other hand, the drug sellers, probably with the perception that self-medication is common in the society, may be more likely to have overstated the adequacy of the information they provided to the women. The implication of this kind of finding is the need for better education both at the provider and community levels, including education about ensuring that services are provided without an assumption about the state or level of knowledge of the client.

Our study had several limitations. First, we selected LGAs purposively and our study is neither representative of Lagos State nor Nigeria. Our complete sample only included drug sellers who admitted selling misoprostol, participated in the KAP survey, agreed to recruit women, and successfully did so. Our sample of women was similarly limited. These drug sellers may thus differ in unmeasured ways from sellers operating within the study areas who do provide MA but did not disclose it to the study team. Second, we only interviewed one person behind the counter of the drug store and when comparing practice with the knowledge there is no guarantee that women received care from the same person we interviewed. Also, there is the possibility of clustering in the sample of women, given that some will have been served by the same pharmacy staff. That said, 61% of PPMV stores had only one staff member so for that subgroup, it is more likely the drug seller information is consistent. Third, we constructed a simple scale to assess the quality of care assigning equal weight to all the questions in each domain with no external validation of the scale or evidence of its impact on outcomes. However, there are currently no published validated scales to evaluate the quality of care for MA amongst drug stores, and the variables included were chosen based on the WHO guidelines for MA provision to reflect recommended practice during the study.

Despite the relatively low quality of care scores and the knowledge-practice gap, our previous publication (16) shows that the majority of our sample of women were able to complete their abortions using the MA purchased from these drug sellers, and very few experienced any postabortion complications. The risk of severe complications and death from unsafe abortions has reduced considerably in many contexts thanks to the availability of MA. Drug sellers are likely to continue to play a significant role in expanding access to MA and other SRH services for women. A recent retrospective survey in Nigeria among medicine vendors that provided MA services for 4,924 clients showed that more than two-thirds of the respondents would prefer to visit these outlets for similar services and other health decisions in the nearest future. As such, PPMVs and Pharmacies are integral for the improvement and expansion of access to medication abortion, particularly those in restrictive contexts, areas with limited physical access to healthcare where telehealth has not yet taken off, and in the context of health emergencies such as the recent COVID-19 pandemic.

Drug sellers' knowledge about the correct usage of the medications and ability to convey key information to women seeking their services is crucial to ensuring women can safely and effectively self-manage their medication abortions. Therefore, on-the-job training for these kinds of frontline providers is an important harm-reduction mechanism to reduce the risk of morbidity and mortality due to unsafe abortions. It is also critical to facilitate independent access to clearly written information on what to expect when using MA, how to identify a complication and seek post-abortion care if necessary, and how to access post-abortion counseling in local languages to support women on this self-management pathway. However, studies have shown that such training for pharmacy and PPMV staff may not yield optimal results due to the high mobility and turnover of staff (1, 32). Also, it is not feasible to scale up trainings to the national level in many developing countries because of the large number drug sellers (33, 34).

## Program/policy implication

The COVID-19 experience and its effect on sexual and reproductive health behavior suggest the likelihood of increasing unwanted pregnancies and demand for MA in Nigeria. It is expected that drug sellers will continue to play significant roles in the provision of misoprostol for abortion, particularly during health emergencies. This highlights the need to promote their skills and experience with misoprostol service provision as a harm reduction strategy.

## Data availability statement

The raw data supporting the conclusions of this article will be made available by the authors, without undue reservation.

## Ethics statement

The studies involving human participants were reviewed and approved by the National Health Research Ethics Committee in Nigeria and the Institutional Review Board of Guttmacher Institute, U.S.A. approved the study. The patients/participants provided their written informed consent to participate in this study.

## Author contributions

OO, AB, and AA conceptualized the research idea and developed the research plan. AA drafted the introduction and discussion. OO and TE drafted the methodology, conducted the data analysis, and wrote the results. All authors contributed to the article and approved the submitted version.

## Funding

This article was made possible by a grant from the Dutch Ministry of Foreign Affairs (#4000000282).

## Conflict of interest

Author OO was employed by Vital Strategies. The remaining authors declare that the research was conducted in the absence of any commercial or financial relationships that could be construed as a potential conflict of interest.

## Publisher's note

All claims expressed in this article are solely those of the authors and do not necessarily represent those of their affiliated organizations, or those of the publisher, the editors and the reviewers. Any product that may be evaluated in this article, or claim that may be made by its manufacturer, is not guaranteed or endorsed by the publisher.

## Author disclaimer

The views expressed are those of the authors and do not necessarily reflect the positions and policies of the donors.
