# Supplementary material for: Quality of information offered to women by drug sellers providing medical abortion in Nigeria: Evidence from providers and their clients
Source: Front Glob Womens Health. 2022 Aug 17;3:899662. doi: 10.3389/fgwh.2022.899662 (PMC9428275; doi:10.3389/fgwh.2022.899662)
Supplement: Supplementary file 1 [file Table_1.pdf]

Appendix Table 1: Quality of care indicators and associated questions from the surveys

Construct/Indicator	The question in woman's tool	The question in drug sellers' tool
**Technical competence**		
Pregnancy confirmation<br>Ask GA/LMP	Did the seller ask you the following?<br>- When you had your last menstrual<br>- If you took a pregnancy test	Before selling a client medication to terminate an unwanted pregnancy/regulate a period, what questions should a seller ask?<br>- Gestational age<br>- pregnancy test/symptoms of pregnancy<br>Knew correct Gestational Age calculation
Clinical: Dose	Aggregated Dosage variable	What is the appropriate dose of misoprostol for abortion for a woman who is in her first trimester of pregnancy?<br>(Answer- 800 µg or greater Buccal, vaginal, or sublingual)<br><br>What is the appropriate dose of misoprostol for abortion for a woman who is in her second trimester of pregnancy?<br>(Answer- 400 µg Buccal, vaginal, or sublingual every three hours (if the timing is not available just use 400))<br><br>What is the appropriate dose of mifepristone/misoprostol in the combi pack for a woman who is in her **first trimester** of pregnancy?<br>(Answer- mifepristone 200 mg orally once, 800 µg Buccal, vaginal, or sublingual)<br><br>What is the appropriate dose of mifepristone/misoprostol in the combi pack for a woman who is in her second trimester of pregnancy?<br>(Answer- mifepristone 200 mg orally once, 400 µg Buccal, vaginal, or sublingual)

		Scored 1 if all 4 are correct.
Clinical: Route of administration	Aggregated route variable	What are the routes via which a woman can use misoprostol for abortion?<br>- buccal<br>- vaginal<br>- sublingual
**<u>Information given to client</u>**		
Give information	Did the drug seller tell you the name of the medicine you took to bring back your period/end pregnancy?<br><br>Did the drug seller tell you the name of this particular medicine?	Do you provide clients seeking medication to terminate an unwanted pregnancy or restore a missed period with information about how to use the medications?
Dosage/route subjective	Did the seller give you instructions on how to take this particular tablet?	When selling a client medication to terminate an unwanted pregnancy/bring back a period, what information should a seller give them?<br>- Dosage<br>- How to take tablets<br><br>*Must select either of both options to get a point*
what she is likely to experience (e.g. menstrual-like cramps, pain, and bleeding), risks and complications associated with the abortion method	What did the drug-seller tell you to expect after taking the tablets? / What did the drug-seller tell your friend you should expect after taking the tablets?<br>- Pill effect: Bleeding<br>- Pill effect: Cramping<br>- Pill effect: Fever/chills<br><br>(Give each of these one point- bleeding, cramping, fever/chills and score 1 if 3/3)	Medical abortion drugs sometimes have complications that may require immediate medical attention. Can you give examples of such warning signs?<br>- Heavy vaginal bleeding that causes you to feel weak<br>- Severe lower abdominal pain<br>- High fever
How to confirm termination is complete		Do you tell clients how to confirm she is no longer pregnant after using the medicine you dispense?<br>*(half point for yes)*<br><br>How do you tell them to confirm they are no longer pregnant?<br>- Pregnancy test<br>- Ultrasound scan<br>- To lookout for an expelled fetus or products of conception

		- Her pregnancy symptoms will disappear<br>- She will get her period<br><br>*(half point if they say 1/2/3/4/5)*
How to address complications		What actions do you recommend these clients take to address their complications?<br>- Seek care at a public hospital or clinic<br>- Seek care at a private hospital or clinic<br>- Seek care at any facility of her choice<br>- Seek care at the nearest health care provider to her home<br><br>*(must say any of the options to get a point)*
**Client experience**		
Pain relief information	Did the seller instruct you to take anything for pain?	

Appendix Table 2: QoC scores at the levels of indicators and domains from drug sellers' and women's perspectives

	**Drug Sellers**				**Women**		
	**Pharmacy**	**PPMV**	**Total**		**Pharmacy**	**PPMV**	**Total**
	**N=70**	**N=56**	**N=126**		**N=153**	**N=233**	**N=386**
	**Mean ± SD**	**Mean ± SD**	**Mean ± SD**		**Mean ± SD**	**Mean ± SD**	**Mean ± SD**
Pregnancy Confirmation	0.43 ± 0.29	0.45 ± 0.26	0.44 ± 0.28		0.57 ± 0.46	0.85 ± 0.32	0.73 ± 0.41
Asked about last menstrual period	0.54 ± 0.50	0.57 ± 0.50	0.56 ± 0.50	Was asked about last menstrual period	0.58 ± 0.50	0.88 ± 0.33	0.76 ± 0.43
Asked about pregnancy test	0.06 ± 0.23	0.02 ± 0.13	0.04 ± 0.20	Was asked about pregnancy test	0.56 ± 0.50	0.81 ± 0.39	0.71 ± 0.45
Knew correct GA calculation	0.70 ± 0.46	0.77 ± 0.43	0.73 ± 0.45				
Dosage First Trimester	0.10 ± 0.22	0.12 ± 0.21	0.11 ± 0.22	Was given an optimal dosage of misoprostol	0.53 ± 0.50	0.58 ± 0.49	0.56 ± 0.50
Prescribed optimal dosage of miso for women in 1st trimester	0.19 ± 0.39	0.23 ± 0.43	0.21 ± 0.41				
Prescribed optimal dosage of miso for women in 2nd trimester	0.00 ± 0.00	0.02 ± 0.13	0.01 ± 0.09				
Dosage Second Trimester	0.00 ± 0.00	0.01 ± 0.07	0.00 ± 0.04				
Prescribed optimal dosage of mife/miso in combi pack for women their 1st trimester	0.01 ± 0.12	0.00 ± 0.00	0.01 ± 0.09				
Prescribed optimal dosage of mife/miso in combi pack for women their 2nd trimester	0.00 ± 0.00	0.00 ± 0.00	0.00 ± 0.00				
Route of administration	0.50 ± 0.26	0.50 ± 0.24	0.50 ± 0.25	Was told an optimal route of misoprostol administration	0.22 ± 0.41	0.16 ± 0.37	0.18 ± 0.39

Buccal	0.13 ± 0.34	0.09 ± 0.29	0.11 ± 0.32				
Vaginal	0.56 ± 0.5	0.48 ± 0.50	0.52 ± 0.5				
Sublingual	0.83 ± 0.38	0.93 ± 0.26	0.87 ± 0.33				
Clinical: Appropriate GA to use drugs							
Knows appropriate GA to use drugs	0.74 ± 0.44	0.84 ± 0.37	0.79 ± 0.41				
TechnicalCompetence (All varriables)	1.78 ± 0.85	1.92 ± 0.74	1.84 ± 0.81		1.31 ± 0.71	1.59 ± 0.71	1.48 ± 0.73
TechnicalCompetence_scaled (All varriables)	0.36 ± 0.17	0.38 ± 0.15	0.37 ± 0.16		0.44 ± 0.24	0.53 ± 0.24	0.49 ± 0.24
TechnicalCompetence (Matching variables)	0.90 ± 0.54	0.92 ± 0.51	0.91 ± 0.52				
TechnicalCompetence_scaled (Matching variables)	0.23 ± 0.13	0.23 ± 0.13	0.23 ± 0.13				
Give information							
Provide clients with information about how to use the medications	0.81 ± 0.39	0.98 ± 0.13	0.89 ± 0.32	Was told the name of the medicine by drug seller	0.13 ± 0.34	0.25 ± 0.43	0.20 ± 0.40
Dosage/Route instructions	0.73 ± 0.41	0.84 ± 0.32	0.78 ± 0.38	was instructed on how to take tablet	0.59 ± 0.49	0.65 ± 0.48	0.63 ± 0.48
Dosage	0.73 ± 0.45	0.82 ± 0.39	0.77 ± 0.42				
Route	0.73 ± 0.45	0.86 ± 0.35	0.79 ± 0.41				
Potential Experience, Risks and Complications	0.52 ± 0.29	0.54 ± 0.33	0.53 ± 0.31		0.32 ± 0.32	0.36 ± 0.27	0.35 ± 0.29
Heavy vaginal bleeding that causes you to feel weak	0.90 ± 0.30	0.77 ± 0.43	0.84 ± 0.37	Bleeding	0.55 ± 0.50	0.73 ± 0.45	0.66 ± 0.47

Severe lower abdominal pain	0.43 ± 0.50	0.52 ± 0.50	0.47 ± 0.50	Cramping	0.37 ± 0.49	0.32 ± 0.47	0.34 ± 0.47
Fever	0.23 ± 0.42	0.32 ± 0.47	0.27 ± 0.45	Fever/Chills	0.05 ± 0.21	0.04 ± 0.19	0.04 ± 0.20
Confirm termination	0.47 ± 0.50	0.53 ± 0.50	0.50 ± 0.50				
Pregnancy test	0.43 ± 0.50	0.46 ± 0.50	0.44 ± 0.50				
Ultrasound scan	0.17 ± 0.38	0.09 ± 0.29	0.13 ± 0.34				
To lookout for an expelled fetus or products of conception	0.06 ± 0.23	0.13 ± 0.33	0.09 ± 0.28				
Her pregnancy symptoms will disappear	0.04 ± 0.20	0.20 ± 0.40	0.11 ± 0.32				
She will get her period	0.11 ± 0.32	0.16 ± 0.37	0.13 ± 0.34				
Addressing complications	0.26 ± 0.44	0.20 ± 0.40	0.23 ± 0.42				
Seek care at a public hospital or clinic	0.17 ± 0.38	0.07 ± 0.26	0.13 ± 0.33				
Seek care at a private hospital or clinic	0.04 ± 0.20	0.04 ± 0.19	0.04 ± 0.20				
Seek care at any facility of her choice	0.04 ± 0.20	0.07 ± 0.26	0.06 ± 0.23				
Seek care at the nearest health care provider to her home	0.03 ± 0.17	0.04 ± 0.19	0.03 ± 0.18				
InformationToClients (All variables)	2.79 ± 1.23	3.08 ± 0.96	2.92 ± 1.12		1.05 ± 0.74	1.26 ± 0.61	1.18 ± 0.67
InformationToClients_scaled (All variables)	0.56 ± 0.25	0.62 ± 0.19	0.58 ± 0.22		0.35 ± 0.25	0.42 ± 0.20	0.39 ± 0.22
InformationToClients (Matching variables)	2.06 ± 0.75	2.36 ± 0.52	2.19 ± 0.67				

<u>InformationToClients_scaled (Matching variables)</u>	0.69 ± 0.25	0.79 ± 0.17	0.73 ± 0.22				
					0.33 ± 0.47	0.25 ± 0.43	0.28 ± 0.45
<u>TOTAL QoC (All Variables)</u>	0.46 ± 0.18	0.50 ± 0.15	0.48 ± 0.17		0.37 ± 0.24	0.40 ± 0.19	0.39 ± 0.21
<u>TOTAL QoC (Matching Variables)</u>	0.46 ± 0.17	0.51 ± 0.12	0.48 ± 0.15				

Appendix Table 3: Overall QoC scores from women's perspectives by their characteristics

Characteristic	Index N=386	F	Prob>F
**Age**			
18-24	0.37	0.49	0.783
25-29	0.38		
30-34	0.41		
35-39	0.40		
40-44	0.37		
45-49	0.29		
**Parity**			
no parity information	0.38	1.00	0.318
no children	0.41		
1-2 children	0.39		
3+ children	0.40		
**Highest Level of Education Completed**			
No schooling or incomplete primary	0.30	0.30	0.823
Primary/Junior secondary school	0.38		
Senior secondary school	0.38		
Some higher education (or more)	0.40		
**Employment Status**			
Work outside the home for pay	0.36	4.04	0.003
Family business or subsistence farm	0.39		
Housewife	0.45		
Student	0.48		
Unemployed	0.38		
**Marital status**			
Currently married or cohabiting	0.39	0.21	0.808
Separated/divorced/widowed	0.41		
Never married/never cohabited	0.38		
**Has previously attempted to end a prior pregnancy**			
No	0.39	0.92	0.339
Yes	0.34		
**Type of pregnancy test taken to confirm pregnancy**			
Confirmation via a test with a doctor			
No	0.38	1.89	0.171
Yes	0.42		
Confirmation via test at a laboratory			
No	0.39	0.79	0.376
Yes	0.36		
Self-administered urine test			
No	0.39	0.06	0.803
Yes	0.39		
